# Supplementary material for: Transcriptome Alterations Caused by Social Defeat Stress of Various Durations in Mice and Its Relevance to Depression and Posttraumatic Stress Disorder in Humans: A Meta-Analysis
Source: Int J Mol Sci. 2022 Nov 9;23(22):13792. doi: 10.3390/ijms232213792 (PMC9698544; doi:10.3390/ijms232213792)
Supplement: Supplementary file 1 [file ijms-23-13792-s001.zip › Supplementary Table S1.pdf]

Supplementary Table S1. A comparison of DEGs between original studies and our reanalysis

Raw data project ID	Alignment Method	Differential expression analysis	Groups	Number of DEGs (FPKM>0.1)	Threshold	Number of reanalyzed DEGs (FPKM>0.1, p.val<0.05)	Common DEGs	Match between new DEGs and original DEGs
GSE146845	STAR aligner	edgeR	SDS	2171	p.val < 0.05	2746	1766	81%
GSE89692	Tophat2, HTSeq-counts	DESeq2	SDS	121	p.val < 0.05 + log_2 FC, LFC > |0.3875|	684	59	49%
GSE72343	Tophat	Cuffdiff	SDS susceptibility	213	p.val <0.05 + log_2 FC, LFC > |0.3875|	2279	134	63%
			resilience toward SDS	291			170	58%
GSE109315 (C57BL/6)	STARv 2.5.0c + HTSeq v0.6.1	limma eBayes	SDS susceptibility	561	Top 300 upregulated and top 300 downregulated genes as ranked by $-log_{10}(p)*\|logFC\|$	1083	155	28%
			resilience toward SDS	550			124	23%
PRJNA323485	TopHat version 2.0.4	DESeq2	SDS 10 days	391	p_{adj} <0.05	2478	361	92%
			SDS 30 days	27		2813	27	100%

Abbreviations. FC: fold change.
