# Supplementary figures and images for: Transcriptome Alterations Caused by Social Defeat Stress of Various Durations in Mice and Its Relevance to Depression and Posttraumatic Stress Disorder in Humans: A Meta-Analysis
Source: Int J Mol Sci. 2022 Nov 9;23(22):13792. doi: 10.3390/ijms232213792 (PMC9698544; doi:10.3390/ijms232213792)

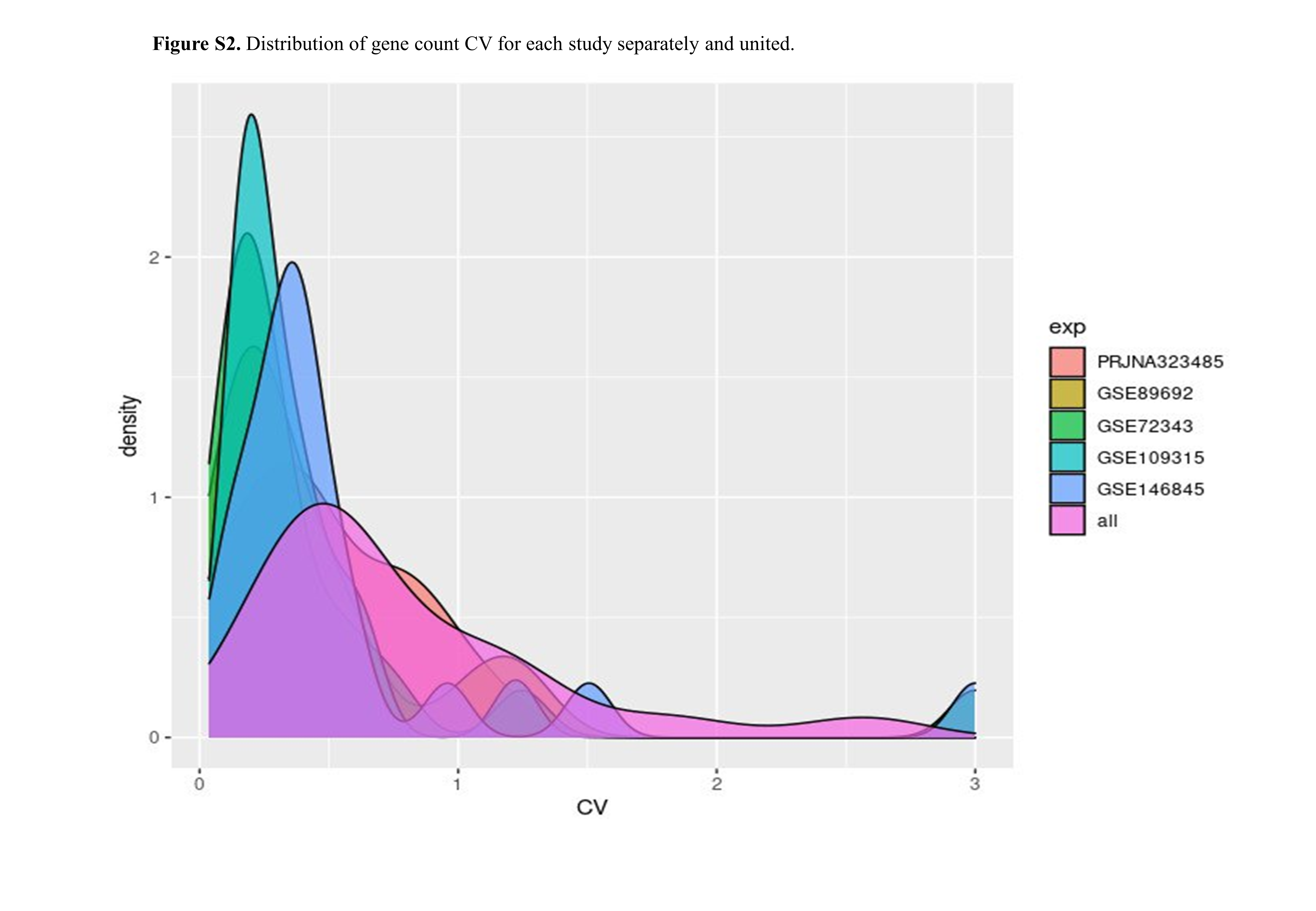

Supplement: Supplementary file 1 [file ijms-23-13792-s001.zip › Figure S2.tif]

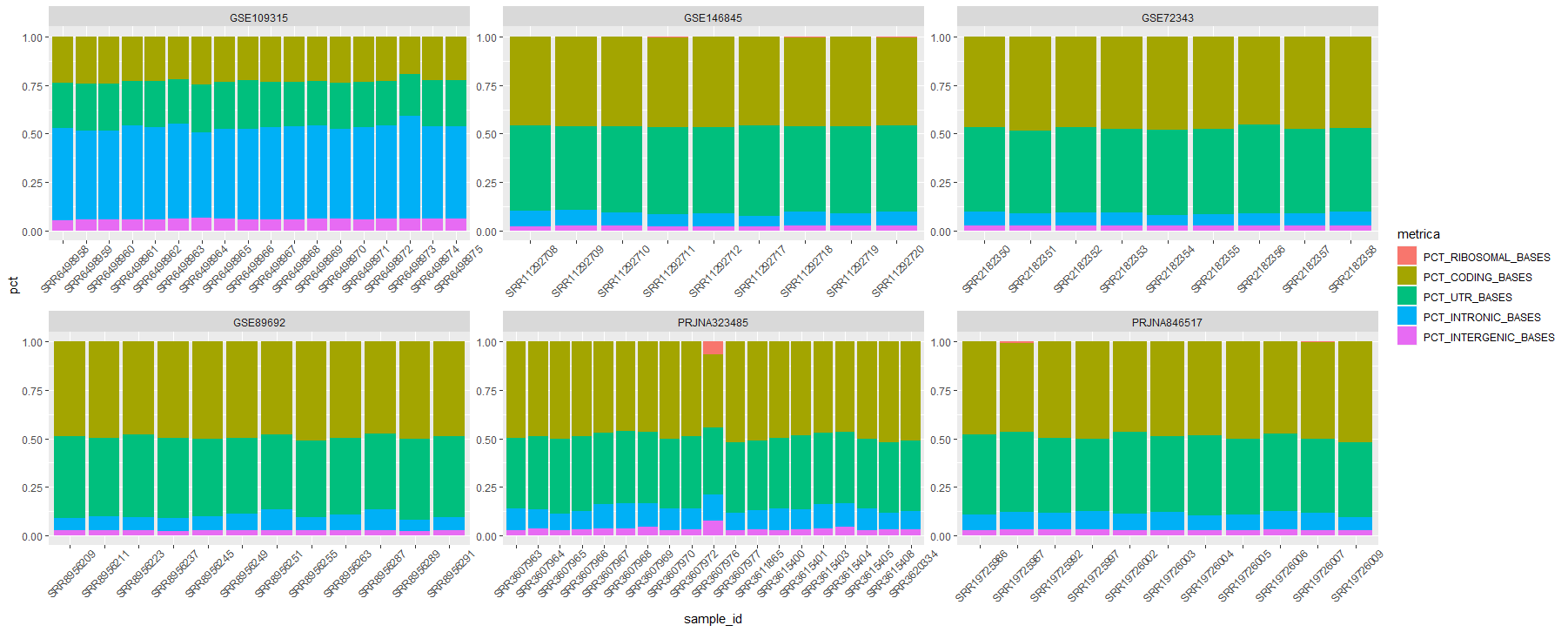

Supplement: Supplementary file 1 [file ijms-23-13792-s001.zip › Supplementary Figure S1.png]
